# Blood-Brain Barrier Permeability of Normal Appearing White Matter in Relapsing-Remitting Multiple Sclerosis

**DOI:** 10.1371/journal.pone.0056375

**Published:** 2013-02-18

**Authors:** Henrik Lund, Martin Krakauer, Arnold Skimminge, Finn Sellebjerg, Ellen Garde, Hartwig R. Siebner, Olaf B. Paulson, Dan Hesse, Lars G. Hanson

**Affiliations:** 1 Danish Research Centre for Magnetic Resonance, Copenhagen University Hospital, Hvidovre, Denmark; 2 Department of Neurology, Danish Multiple Sclerosis Research Center, Copenhagen University Hospital, Rigshospitalet, Denmark; 3 Neurobiology Research Unit, Copenhagen University Hospital, Rigshospitalet, Denmark; 4 Biomedical Engineering Group, Technical University of Denmark, Kgs. Lyngby, Denmark; Beijing Normal University, China

## Abstract

**Background:**

Multiple sclerosis (MS) affects the integrity of the blood-brain barrier (BBB). Contrast-enhanced T1 weighted magnetic resonance imaging (MRI) is widely used to characterize location and extent of BBB disruptions in focal MS lesions. We employed quantitative T1 measurements before and after the intravenous injection of a paramagnetic contrast agent to assess BBB permeability in the normal appearing white matter (NAWM) in patients with relapsing-remitting MS (RR-MS).

**Methodology/Principal Findings:**

Fifty-nine patients (38 females) with RR-MS undergoing immunomodulatory treatment and nine healthy controls (4 females) underwent quantitative T1 measurements at 3 tesla before and after injection of a paramagnetic contrast agent (0.2 mmol/kg Gd-DTPA). Mean T1 values were calculated for NAWM in patients and total cerebral white matter in healthy subjects for the T1 measurements before and after injection of Gd-DTPA. The pre-injection baseline T1 of NAWM (945±55 [SD] ms) was prolonged in RR-MS relative to healthy controls (903±23 ms, p = 0.028). Gd-DTPA injection shortened T1 to a similar extent in both groups. Mean T1 of NAWM was 866±47 ms in the NAWM of RR-MS patients and 824±13 ms in the white matter of healthy controls. The regional variability of T1 values expressed as the coefficient of variation (CV) was comparable between the two groups at baseline, but not after injection of the contrast agent. After intravenous Gd-DTPA injection, T1 values in NAWM were more variable in RR-MS patients (CV = 0.198±0.046) compared to cerebral white matter of healthy controls (CV = 0.166±0.018, p = 0.046).

**Conclusions/Significance:**

We found no evidence of a global BBB disruption within the NAWM of RR-MS patients undergoing immunomodulatory treatment. However, the increased variation of T1 values in NAWM after intravenous Gd-DTPA injection points to an increased regional inhomogeneity of BBB function in NAWM in relapsing-remitting MS.

## Introduction

Multiple sclerosis (MS) is an autoimmune disease affecting the central nervous system and is one of the major neurologic diseases in the Western world. MS disrupts the integrity of the blood-brain barrier (BBB) enabling active components of the immune system to enter the brain parenchyma [1]. Subsequently a cascade of immunological events [2] leads to the characteristic lesions seen on magnetic resonance images (MRI) of the brain or spinal cord [3]. There is evidence that this disruption of the BBB is of major relevance in the early dynamics of the focal MS lesions [2]. Whether early disruption of the BBB allows for lesions to develop or whether an early inflammatory event causes the BBB disruption is still a matter of debate as the two phenomena seems to occur in parallel [4].

The local disruption of the BBB can be captured with T1 weighted MRI after intravenous injection of a Gadolinium (Gd) chelated paramagnetic contrast agent [5]. The contrast agent has a shortening effect on the longitudinal relaxation time (T1) of neighbouring water protons. Since the contrast agent penetrates the disrupted BBB and accumulates locally in the affected brain parenchyma it causes an increase in signal intensity (i.e., contrast enhancement) on T1 weighted images.

In a clinical setting, contrast-enhanced T1 weighted MRI is particularly useful to detect focal BBB disruption in active MS lesions. This virtue makes contrast-enhanced T1 weighted MRI a sensitive tool to detect dissemination of the disease process in space and time, which is essential for making an early diagnosis of MS [6]. The number and location of lesions [6] or their total volume [7] is often used as a surrogate marker for disease severity even though they show only moderate correlations with both cognitive impairments [8], [9], [10] and neurological disabilities [9], [11]. This modest correlation might be due to unknown pathological severity of the lesions and to the fact that symptoms also depend on the location of the lesions. Further, as demonstrated by various quantitative MRI techniques, there is converging evidence that the pathological process extends into the normal appearing white matter (NAWM). Diffusion MRI revealed altered fiber structure in the NAWM [12], while proton-magnetic resonance spectroscopy (MRS) provided changes in the spectrum compatible with axonal loss [13]. MRI has also yielded a reduction of magnetization transfer ratios which has been attributed to a destruction of macromolecules [14]. Histopathological studies have shown increased levels of gliosis, demyelination and cellular infiltration [15], [16] which might be the histological correlate for the alterations revealed by MRI and MRS. Recent studies, however, suggest that changes in the NAWM may primarily be evident in patients with progressive MS [16], [17].

The structural changes in NAWM have been shown to correlate with clinical disability [9], [18], [19], [20]. This converging evidence suggests that pathological processes within the NAWM are contributing to the clinical disability, yet it is unclear to what extent the BBB is disrupted in NAWM and whether such disruption has impact on the functional impairment of MS patients. In this context, it is relevant to recall that the majority of MS patients receive immunomodulatory treatment, which might stabilize BBB integrity in NAWM. A disruption of the BBB in NAWM has been demonstrated in a histopathological study [21]. However, this is in contrast with an *in vivo* MRI study which included 33 patients with either RR-MS or secondary or primary progressive MS and five healthy control subjects [22]. In that study, the quantification of contrast enhancement following the intravenous administration of 0.3 mmol/kg Gd-DTPA yielded no evidence for a disruption of BBB integrity in the NAWM [22].

In general, intravenous injection of a paramagnetic contrast agent shortens T1 throughout the brain – including the white matter of healthy controls [23]. This effect is therefore not specifically related to pathology. Using a new approach to quantitatively measure T1 values, we aimed at assessing differences in contrast agent accumulation in the NAWM of patients with relapsing-remitting MS (RR-MS) and cerebral white matter of healthy age-matched controls. We hypothesized that compared to healthy controls a diffuse disruption of the BBB in MS patients would cause a greater change in the mean T1 value.

## Results

**Figure 1:**
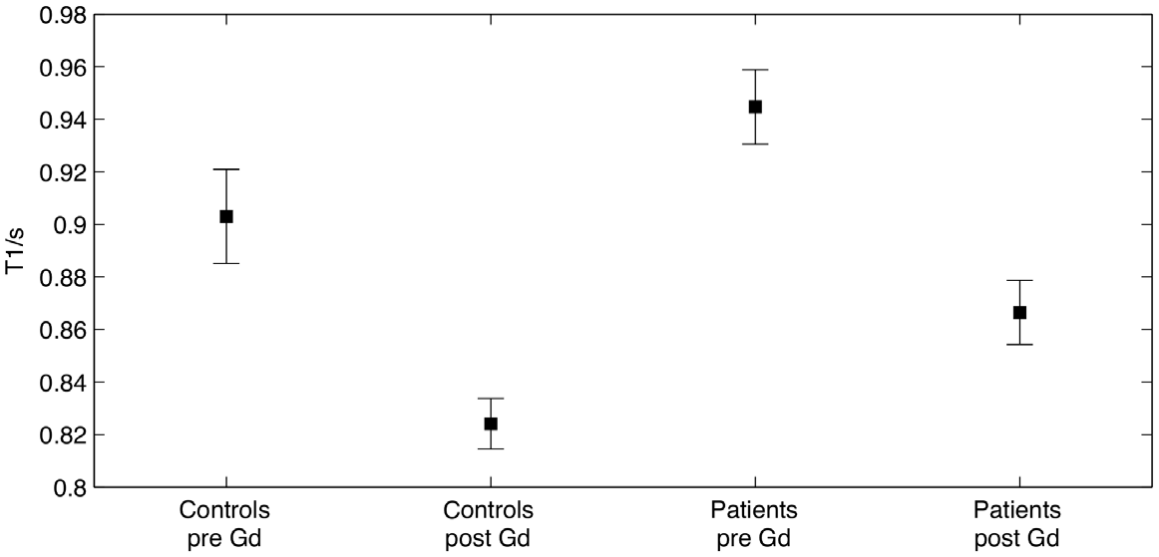
T1 values of the healthy controls and the patients. The plot shows mean T1 (s) and 95% confidence intervals. The injection of contrast agent significantly reduced T1 for both patients and controls. Compared to healthy white matter, the T1 value in NAWM of patients was higher both before and after the injection of contrast agent.

**Figure 2:**
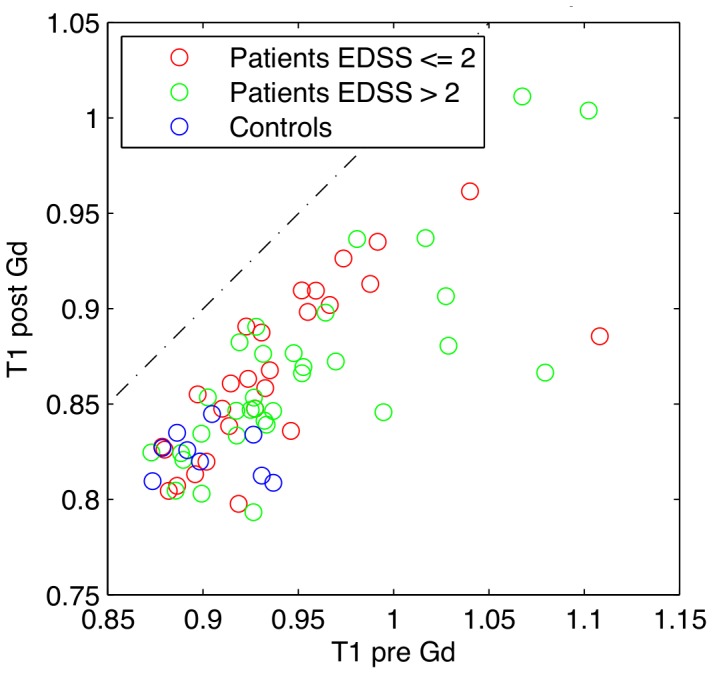
T1 values before and after the injection of contrast agent. For each healthy control and each patient, the mean T1 of the white matter and NAWM, respectively was calculated. Each subject is thus represented by a marker in the corresponding position. All individual subjects had lower T1 values after the injection of contrast agent (*i.e.* all markers are plotted below the line representing *y = x*).

**Figure 3:**
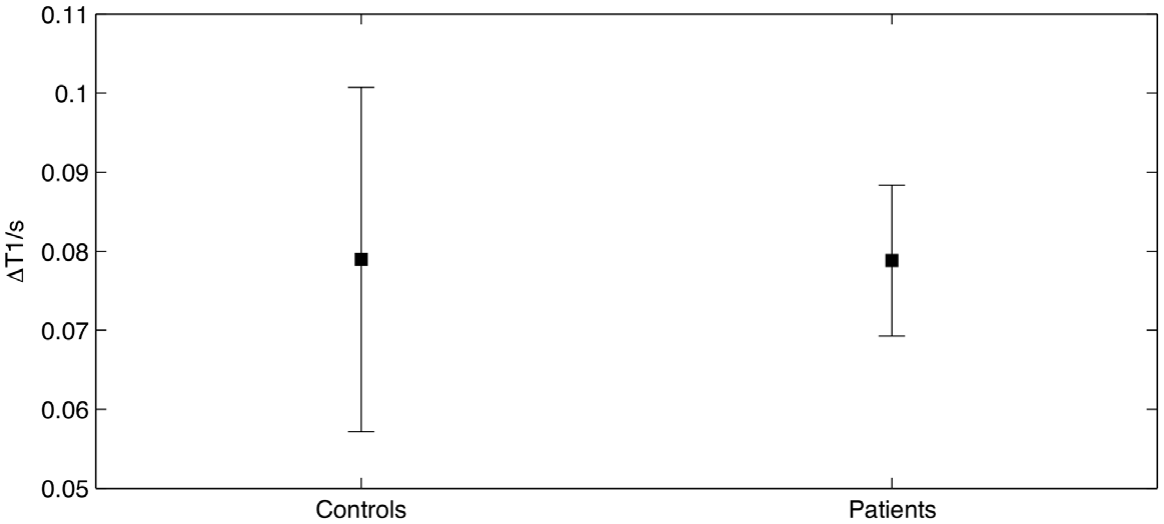
Change in T1 values caused by the injection of the contrast agent. The plot shows the mean change in T1 and 95% confidence intervals. We found no difference between the two groups when evaluating the effect of contrast agent on the T1 value.

Quantitative multi-point T1 measurements revealed significantly higher mean T1 values in NAWM in patients with RR-MS compared to white matter in healthy controls, both before (t = 2.255, p = 0.028, df = 67) and after (t = 2.676, p = 0.009, df = 66) the injection of contrast agent (Figure 1, Table 1). The NAWM in MS patients (t = 16.51, p<<0.001) and the cerebral white matter in healthy controls (t = 8.36, p<<0.001) showed a significant decrease in the mean T1 value after injection of 0.2 mmol/kg Gd-DTPA (Figures 2 and 3). The reduction in mean T1 value was comparable between patients and healthy controls. Accordingly, the ANOVA showed a main effect of group (F = 5.625, p = 0.021) and “time” (before vs. after Gd-DTPA injection) (F = 1,140, p<<0.001) but no interaction between group and time.

**Table 1:** Group data for healthy controls and patients with relapsing-remitting multiple sclerosis (RR-MS).

	Healthy control subjects	Patients with RR-MS
Number (male/female)	9 (5/4)	59 (21/38)
Age	34.5±9.4 years	38.2±8.1 years
Mean T1 (baseline)	903±23 ms	945±55 ms
Mean T1 (Gd-DTPA)	824±13 ms	866±47 ms
Coefficient of variation (baseline)	0.178±0.088	0.160±0.035
Coefficient of variation (Gd-DTPA)	0.166±0.018	0.198±0.046
Median EDSS score	N/A	2.5
Number of voxels in analysis	898.4±327.1	1410.1±260.0
Lesion volumes	N/A	11.3±13.5 ml

For each healthy control and RR-MS patient, the mean and coefficient of variation of T1 values were calculated for measurements before and after intravenous injection of 0.2 mmol/kg Gd-DTPA. Only the cerebral white matter of healthy controls and normally appearing white matter (NAWM) of patients were evaluated. Data are given as mean ± onefold SD.

The mean T1 values of each participant at pre-injection baseline were plotted against the mean T1 values after injection of contrast agent (Figure 2). It is evident from this plot that the injection of contrast agent consistently reduced the mean T1 value in both healthy controls and patients. The data of the nine healthy control subjects showed limited variation as all data points clustered in the lower left corner (Figure 2). In contrast, the T1 data of the patient group was more scattered indicating stronger between-subjects variability in MS patients. The scatter plot of the T1 values before and after contrast agent injection did not reveal a clustered distribution of the data that would allow a delineation of specific subgroups (Figure 2). No significant differences in the response to Gd-DTPA injection could be detected between MS patients with an expanded disability status scale (EDSS) score ≤2 and patients with an EDSS score of >2. Accordingly, we did not find any correlations between EDSS or age and T1 values before or after injection of contrast agent. Neither did EDSS or age correlate with the change in T1 caused by injection of contrast agent (not shown).

**Figure 4:**
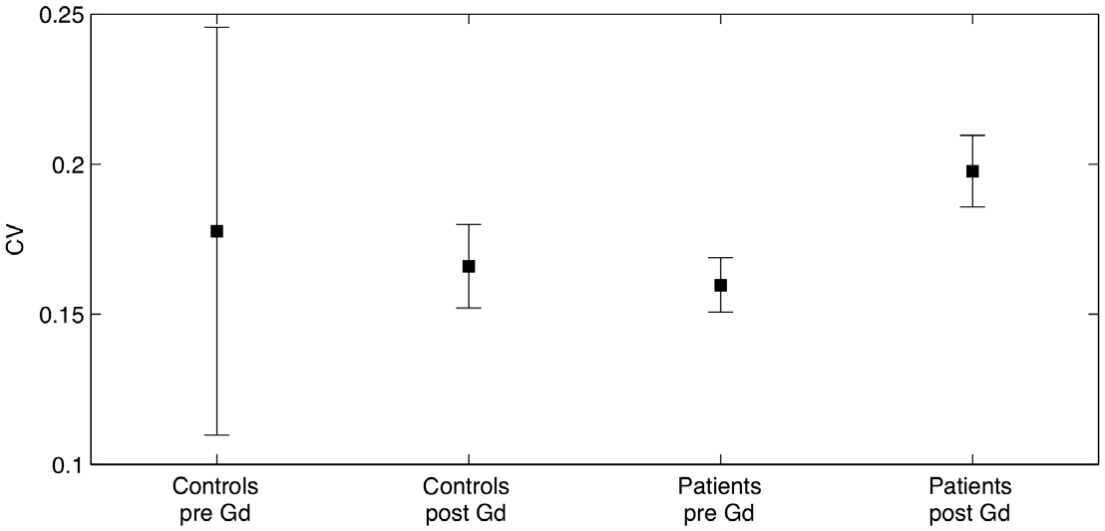
Coefficient of variation of the healthy controls and the patients. The plot shows mean coefficient of variation and 95% confidence intervals. After the injection of contrast agent the mean CV for patients was significantly larger than that of controls. This was not the case prior to the injection of contrast agent.

**Figure 5:**
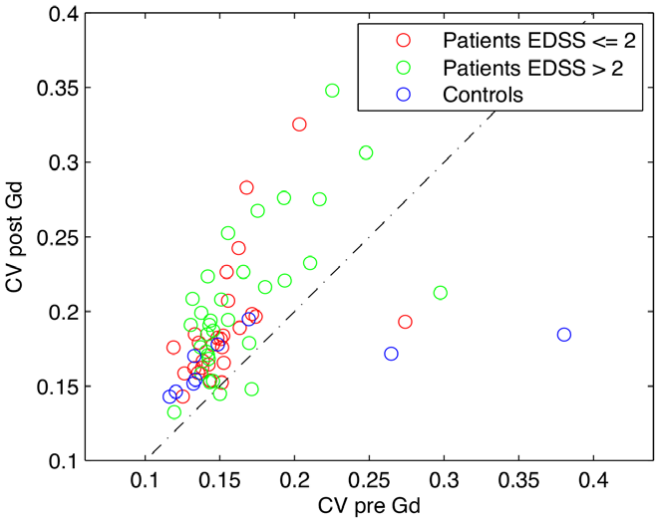
Coefficient of variation before and after the injection of contrast agent. For each healthy control and each patient, the coefficient of variation (CV) of the white matter and NAWM, respectively was calculated. Each subject is thus represented by a marker in the corresponding position (the dotted line represents *y = x*). It seems that the CV of the patients are generally increased after the injection of the contrast agent.

If MS impaired the BBB integrity only in some spots of the NAWM, one might expect that MS patients would show more heterogeneous T1 values within NAWM after contrast agent injection. Therefore, we also assessed the coefficient of variation of T1 values in NAWM at baseline and after intravenous Gd-DTPA injection (Figures 4 and 5, Table 1). The regional variability of T1 values as expressed by the coefficient of variation was comparable between the two groups at baseline (t = 0.60, p = 0.56) but not after injection of the contrast agent (t = 2.04, p = 0.046). Accordingly, a two-factorial ANOVA with the between-subject factor “group” (controls vs patients) and the within-subject factor “time” (before vs. after Gd-DTPA injection) did not reveal a main effect of group (F = 0.007, p = 0.934). However, we did see a main effect of time (F = 34.2, p<<0.001) as well as an interaction between group and time (F = 9.25, p = 0.003).

## Discussion

This MRI study yielded three main findings: First, T1 values in NAWM of patients with RR-MS were significantly higher compared to the T1 values of the cerebral white matter in healthy controls. This was the case at baseline as well as after injection of Gd-DTPA. Second, Gd-DTPA injection consistently reduced the mean T1 value in NAWM of patients with RR-MS as well as in white matter of the controls. In contrast to our research hypothesis, the change in T1 value was comparable in size in patients and healthy controls. Third, the heterogeneity of the T1 values after the injection of contrast agent was higher in patients, suggesting a more variable BBB function in the various subregions of the NAWM.

### Structural Changes in the Normal Appearing White Matter in Multiple Sclerosis

Several groups employed quantitative magnetic resonance methods to demonstrate a diffuse involvement of NAWM in MS. Using proton MRS, Sarchielli et al. found reduced levels of N-acetyl-aspartate (NAA) within the NAWM compared to white matter of healthy controls [13]. Since NAA is considered a specific marker of functional neurons, lower NAA levels within the NAWM indicate a reduction in axonal density in this area. Accordingly, Filippi et al. [12] found a significantly higher mean diffusivity in the NAWM of patients compared to white matter of controls using diffusion weighted MRI. The NAWM also displays a reduced magnetization transfer ratio (MTR) [14]. The MTR reflects the transfer of magnetization between protons in macromolecules and protons in surrounding free water and is associated with axonal density of the brain tissue [24], [25]. These neuroimaging data are in agreement with histological findings of increased levels of gliosis, demyelination and cell infiltrations within the NAWM [15], [16]. Together, these reports give evidence of pathological changes within the NAWM at structural as well as at functional levels. Further, the clinical relevance of these NAWM changes is emphasized by their correlations with neuropsychological measures [18], [26].

The quantitative T1 measurements at baseline showed higher mean T1 values in NAWM of patients with RR-MS compared to the T1 values of the cerebral white matter in healthy controls, lending further support to the notion that the structure of the NAWM is altered in MS. Our baseline T1 measurements further revealed that the coefficient of variation of the T1 values in the NAWM did not differ between MS patients and healthy controls. We therefore infer that the increase in T1 values in MS patients is diffuse rather than regional.

The findings are in good agreement with a previous MRI study in which T1 values in NAWM were reported to be increased in MS [27]. A voxel-based MRI study at 1.5 T focused on the spatial pattern of T1 abnormalities in MS. In that study, large parts of the NAWM displayed increased T1 values and the voxels with increased T1 values were distributed throughout the brain without a particular anatomic preference [28]. Another 1.5 T MRI study found significantly higher T1 values in MS patients, but only in the occipital part of the NAWM [29]. It needs to be mentioned though that not all studies have found significantly higher mean T1 values in RR-MS [30].

### Integrity of the Blood-brain Barrier in the NAWM

RR-MS patients showed a consistent reduction of mean T1 value in NAWM after Gd-DTPA injection. This reduction matched the one found in white matter of healthy controls. Accordingly, mean T1 values in NAWM were higher in RR-MS patients than in healthy controls, both at baseline and after contrast agent injection. This is in accordance with the MRI study by Silver et al. [22] in which 12 RR-MS patients also showed no difference in T1 value change after injection of 0.3 mmol/kg Gd-DTPA relative to five healthy controls.

The injection of contrast agent increased the variation of T1 values within the NAWM of patients but not in the cerebral white matter of healthy controls (Figure 5). In other words, the mean decrease in T1 value was normal in NAWM, but local changes in T1 value induced by contrast agent injection were more variable in RR-MS patients. Two mechanisms with opposite effects on the T1 value might account for the increased heterogeneity of T1 values in NAWM after contrast agent injection. While regional disruptions of the BBB may result in an increased amount of GD-DTPA in the tissue and thereby stronger reductions of the T1 value after contrast injection, a reduced blood compartment in NAWM might lead to a decreased amount of Gd-DTPA and thereby attenuate the reduction in T1 value. If these two mechanisms are differentially expressed in the NAWM, this may increase the heterogeneity of T1 values in the presence of a normal reduction in mean T1-values. Whatever the mechanism may be, the increased variability in T1 values after contrast agent injection raises the possibility that BBB dysfunction in the NAWM is locally rather than globally expressed. Further, it is possible that between-group differences were partly concealed because of partial volume effects and inter-slice gaps. We might have observed larger coefficients of variation by reducing the voxel size and eliminating the gaps between consecutive slides. However, smaller voxel sizes would have resulted in a lower signal to noise ratio and/or an increased acquisition time. The regional expression of BBB function in NAWM requires further investigation.

In RR-MS patients, the decrease in mean T1 value was comparable when splitting patients in two groups with an EDSS score ≤2 or >2. This confirms a previous MRI study [22] and speaks against a close correlation between global BBB integrity of the NAWM and clinical impairment in MS.

We also wish to point out that all included patients were on treatment with interferon beta or glatiramer acetate. We therefore cannot exclude the possibility that the natural course of RR-MS might be associated with a BBB dysfunction of NAWM, but that this BBB disruption was reversed by immunomodulatory therapy. Indeed, the fact that patients had been treated with interferon beta and glatiramer acetate could very well reduce the probability of detecting BBB disruptions, as immunomodulatory treatment decreases the number of Gd-enhancing lesions [31], [32]. Further, the immunomodulatory treatment attenuates the natural intermittent inflammatory process of RR-MS patients and hereby reduces the probability to detect subtle disruption of the BBB in NAWM. Therefore, the present study provides no information about BBB dysfuntion that might be observed during the natural course of the disease. However, recruiting drug-naïve patients that do not receive any immunomodulatory treatment would not be realistic in Denmark where practically all patients with MS are treated with immunomodulatory drugs.

### Physiological Considerations

A number of other factors related to the nature of a BBB damage need to be considered when interpreting the present results. First, the early disruption of the BBB in an affected region in MS might be caused by capillary wall adhesion of immunoactive cells, which subsequently penetrate the endothelial cellular layer and enter the cerebral tissue. At a later stage the leaky BBB allows larger, hydrophilic substances like Gd-DTPA (molecular weight: 938 g/mol [33]) to cross. Such passage could take place either through gaps in damaged tight junctions between the endothelial cells or by abnormal pinocytotic activity in the endothelial cells allowing vesicular transport between the luminal and abluminal membrane. Both pinocytosis [34] and abnormal tight junctions [35] have been demonstrated in MS lesions. Although histopathological studies by Kirk et al. [35] found no statistically significant difference between tight junction abnormalities in normal controls and NAWM they did see a trend. The extent to which these mechanisms occur within the NAWM remains to be fully elucidated.

Second, focal tisue damage in demyelinated lesions leads to a secondary loss of axons within the NAWM [36]. Such Wallerian degeneration causes both structural and functional changes of the axons that might contribute to all the above-mentioned measures of diffusion, MTR and NAA levels. On the other hand, it is unlikely that Wallerian degeneration would affect the BBB function which would also be in line with the findings obtained in the present study.

Third, timing of the data acquisition after injection of a contrast agent is perhaps of importance to the reduction in the T1 value. Silver et al. [22] found that within the white matter of healthy controls, the reduction in T1 caused by the injection of contrast agent vanished over the course of approximately 20 minutes. Considering that healthy tissue is expected to have an intact BBB, we would not expect such tissue to show a sustained effect of the contrast agent. Lesions of MS patients, on the other hand, have been shown to exhibit a prolonged enhancement up to an hour [37]. It is likely that the duration of the effect of a contrast agent within the NAWM is somewhere between that for lesions and healthy tissue. A future study evaluating the rate at which T1 returns to its pre-contrast level could reveal differences between NAWM in MS and white matter of healthy controls.

### Methodological Considerations

The STEAM sequence used in the present study gives a relatively fast, quantitative, multi-point T1 measurement robust to inhomogeneity of the scanner’s radiofrequency field (B1 inhomogeneity). Combining the T1 value before and after the injection of a contrast agent by calculating their mean difference on an ROI basis, we obtained a measure of the BBB integrity which is unbiased and potentially more sensitive compared to the regular approach using T1 weighted images. A coregistration of T1 maps before and after contrast injection would result in substantial data degradation since the voxelwise T1 differences between the two scans are very sensitive to motions between scans. Hence, a voxelwise comparison was not performed. However, calculating a coefficient of variation on “delta-T1” values across voxels for each subject might be a better measure to detect higher regional variability of contrast-induced T1 changes in NAWM relative to white matter in healthy controls. We used 0.2 mmol/kg Gd-DTPA (so called “double dose”) theoretically doubling the contrast agent-induced signal change compared to a standard dose [38].

Ideally, measurements of T1 values are independent from the type of sequence or scanner. Often, however, one needs to compromise on acquisition time or validity of the data. For example, T1 measurements performed by varying the inversion time (inversion recovery) typically require a relatively long acquisition time. Alternative methods, based on variation of sequence tip angles or repetition time TR, are limited by the validity of assumed signal equations, which are most often approximate. Further, the latter methods and other fast T1 measurements including the one used in the present study are sensitive to B1 inhomogeneities [39] and valid B1 maps are needed for correction.

Our reported T1 values are estimated from a 30-point measurement using a novel MRI sequence. The performed analysis assumed monoexponential T1 relaxation in each voxel. No evidence of non-monoexponential relaxation at the ROI level was found based on visual inspection of a subset of the fits. Also, an extended model including a normal distribution of relaxation rates was used. It was found to cause overfitting when applied to data at the ROI level partially corrected for B1 inhomogeneity based on an assumption of monoexponential relaxation at the voxel level. This finding is consistent with monoexponential relaxation behavior at the ROI and voxel levels but does not guarantee either. Hence, a B1-corrected monoexponential model was applied at the voxel level. We confer that acquiring fewer data points on the T1 relaxation curve would suffice for a valid T1 estimate. Choosing few points optimally, however, would require T1 values to be known a priori.

We found pre contrast T1 values in the group of healthy controls to be 903 (±23) ms, which is in good agreement with previously reported values [30] but slightly higher than 832 ms as reported by Wansapura et al. [40]. The study by Wansapura et al. did not address the issue of transmit RF field (B1) inhomogeneities that likely imply a spatially dependent bias in T1 estimates.

### Concluding Remarks

BBB disruption is an early and very important event in the development of MS lesions. The assessment of BBB disruption in the NAWM is therefore of major importance to the diagnosis and monitoring of MS and to the validation of new treatment strategies – particularly because early treatment is known to improve the clinical outcome [41]. The presented method is novel and demonstrates a proof-of-principle applied on both patients and controls.

Our quantative T1 measurements showed that RR-MS is associated with diffuse structural abnormalities in NAWM as evidenced by a decrease in T1 values, but this does not include a diffuse disruption of BBB integrity. Since the present study considered the NAWM as a whole, it remains to be explored to what extent RR-MS is associated with more localized BBB disruptions in NAWM and whether such changes can be detected using quantitative and more sensitive contrast enhanced MR techniques. Still our study indicates that localized changes in BBB permeability may be present as we observed an increased coefficient of variation of T1 following Gd-DTPA in NAWM of the patients as compared to white matter in the controls.

## Materials and Methods

### Ethics Statement

The study was conducted in accordance with the Declaration of Helsinki and was approved by The Danish National Committee on Biomedical Research Ethics, Capital Region (KF 01-041/95 and KF 01-258762). Written informed consent was obtained from all participants before they participated in the study.

### Participants

We prospectively acquired MRI data from 59 patients (21 males, 38 females) with definite MS according to the revised McDonald criteria [6] and a disease duration of at least six months. Of the 59 patients, 38 were on treatment with interferon beta and 21 received glatiramer acetate. Patients had a mean age (± standard deviation) of 38.2 (±8.1) years. At the time of MRI examination, patients had a median EDSS [42] score of 2.5, ranging from 0 to 6.0. All patients had a relapsing-remitting disease course. We also studied nine healthy controls (5 males, 4 females) with a mean age of 34.5 (±9.4) years. No abnormalities were detected in the healthy controls.

### Magnetic Resonance Imaging

Scans were performed on a 3.0 tesla whole-body scanner (Trio, Siemens, Erlangen, Germany) using an 8-channel head coil. Prior to the imaging session an intravenous cannula was inserted and connected to a line to avoid re-positioning of the participant after the injection of the contrast agent.

#### Standard structural MRI measurements

A set of whole-brain structural images was acquired before the injection of the contrast agent. This included a Magnetisation Prepared Rapid Acquisition Gradient Echo (MPRAGE) sequence with a repetition time (TR) of 1540 ms, echo time (TE) of 3.9 ms, inversion time (TI) of 800 ms and a 1 mm isotropic resolution (192 slices). We additionally acquired T2 weighted brain images using a double spin echo sequence (TE 115 ms, TR 8.8 s) with an in-plane resolution of 0.7×0.7 mm^2^, a slice thickness of 4 mm, and a refocusing tip angle of 163°. A Fluid Attenuated Inversion Recovery (FLAIR) sequence was also acquired (TE 97 ms, TI 2500 ms and TR 10 s) with an in-plane resolution of 0.7×0.7 mm^2^, a slice thickness of 4 mm, and a refocusing flip angle of 150°.

#### Quantitative T1 measurements

In all subjects, 0.2 mmol/kg Gd-DTPA (Gadovist, Bayer Pharma AG, Germany) were injected through an intravenous line. Quantitative T1 measurements were acquired both before and approximately 20 min after the injection of the contrast agent. For quantitative T1 measurements, we chose a STimulated Echo Acquisition Mode (STEAM) sequence, which enabled us to sample 30 points on the T1 relaxation curve in a whole-brain volume within 25 seconds. Echo Planar Imaging (EPI) and acquisition of trains of stimulated echoes were combined to provide voxel-wise multi-point T1 measurements over many slices in a short time window. This novel approach is time efficient, but the analysis requires care regarding B1 inhomogeneity as addressed below.

**Figure 6:**
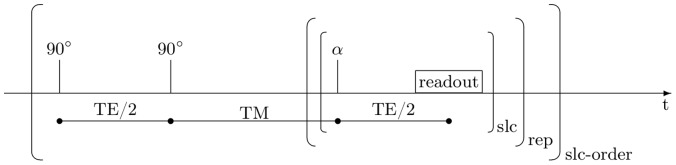
Sequence used for T1 measurements. The applied STEAM sequence used for T1 quantification includes loops over slices (slc), repetitions (rep) and slice orderings (slc-order).

The STEAM sequence corresponded to the sequence described in [43], but diffusion gradients were replaced with shorter spoiler gradients giving minimal diffusion weighting. The applied STEAM sequence starts with two non-selective excitation pulses having tip angles near 90° (Figure 6). The third excitation pulse of the typical STEAM sequence is replaced with nested loops involving repeated slice-selective small-angle excitations (α pulses), each followed by EPI readout. The inner loop (slc) is over slices. The number of repetitions in the outer loop (rep) determines how many points are acquired on the T1 relaxation curves per STEAM preparation. Since the EPI acquisition in different slices are time shifted on the T1 relaxation curves, and in order to measure all slices at the same time points after excitation, the whole sequence is repeated (slc-order) with the excitation order of slices permuted as many times as there are slices.

The parameters for the T1 measurement were: in-plane resolution 3.4×3.4 mm^2^, slice thickness 8 mm (2 mm gaps), field of view 220 mm, 15 slices (whole-brain volume), tip angle α = 15°, TR 1740 ms, TE 40 ms and 30 evenly distributed STEAM mixing times between 11 and 1,661 ms.

#### B1 measurement

Like other fast T1 measurements, the developed T1 sequence is sensitive to the radiofrequency field (B1) inhomogeneity [39], and must therefore be combined with B1 measurements to provide accurate quantification. Such measurements were performed with a variant of the sequence described above, also involving stimulated echo acquisition and echo planar readout as described in [44]. Again, the sequence involved a non-selective STEAM preparation followed by multi-slice excitation and EPI readout. There was only one loop over slices, however. In order to change this known multi-slice STEAM sequence [45] into a B1 measurement [46], a non-selective pulse and appropriate extra spoiling was inserted in the so-called mixing period of the sequence where prepared magnetization is stored longitudinally for a period TM. This extra pulse had a freely selectable tip angle. Since it partially removes the stored longitudinal magnetization in the TM period, the signal exhibits a clean cosine-dependence on the actual tip angle of this non-selective pulse, independent of *e.g.* relaxation, other tip angles, slice profiles and partial volume. This only requires that the magnetization is equally relaxed between sequence repetitions with different tip angles, which is ensured by choosing a long TR. In this way, B1 was measured accurately in 75 seconds. Parameters for the B1 measurement were: in-plane resolution 3.1×3.1 mm^2^, slice thickness 8 mm, field of view 400 mm, 15 slices (covering the entire brain), tip angle 30° (slice selective pulse), TR 10.6 s, TM 11 ms and TE 29 ms. The two initial STEAM tip angles were 80° and the latter 30° to ensure near-complete recovery of the longitudinal magnetization within the TR period. The adjustable tip angle was altered between 0° and 350° in steps of 50°.

The B1 maps showed expected significant deviations between nominal and actual tip angles, reflecting coil and head geometries, and varying smoothly across tissue boundaries. The maps were used to calculate T1 estimates free of B1 inhomogeneity in a single compartment model.

#### Handling B1 inhomogeneity and fitting T1

If no B1 inhomogeneity or partial volume effects were present and the magnetization was fully relaxed before each STEAM preparation, the measured signal would decay exponentially as a function of TM on a timescale depending on the longitudinal relaxation rate and the nominal excitation tip angles. Neither of these conditions is fulfilled, however, and the signal is therefore not easily expressed in closed form. To keep the sequence duration short, the TR was chosen so that not all tissue components had relaxed fully before STEAM preparations. Due to the slice order permutations, this gives rise to small non-trivial deviations from exponential behavior compromising simple fitting as a means of doing T1 estimation. Variation in tip angle furthermore results from B1 inhomogeneity and reliable T1 estimation therefore requires B1 to be known. Both of these effects were accounted for in a T1 estimation performed by modeling a voxelwise fit to the measured signal using the Levenberg-Marquardt algorithm. The spin evolution was modeled from solutions of the Bloch equations for periods of relaxation interrupted by hard pulses of known tip angle. The modeling effectively filtered out additional small signal contributions coming from slice crosstalk. The resulting fits, representing single compartment approximations to the measured signal, provided quantitative T1 maps as well as relative proton density maps (used for spatial normalization).

#### Image pre-processing

Pre-processing in the patient group: The following procedures were performed in each patient. Lesions were identified by simultaneous visual inspection of proton density images, T2 weighted images and FLAIR images. Tissue classified as lesion was marked on the FLAIR image using a semi-automatic thresholding technique with in-house developed software (RIP, http://www.drcmr.dk/software/downloads.html). Tissue classification was performed by a trained observer, blind to any clinical as well as neuropsychological data.

Automated segmentation, co-registration and reslicing were done using SPM5 (http://www.fil.ion.ucl.ac.uk/spm/software/spm5/). The performed tissue segmentation was based on the MPRAGE images and resulted in only three compartments: CSF, gray matter and white matter. Consequently, any voxel in a lesion will falsely be categorized as either of these. However, because lesions were excluded from our analysis of the NAWM such misclassification would not influence our results.

The FLAIR images were coregistered to the MPRAGE using a linear coordinate transformation with 6 parameters (3 translational and 3 rotational) and the resulting transformation matrix applied to the lesion masks, bringing these into native space of the MPRAGE image. A NAWM mask was constructed by excluding the lesion masks from the white matter mask followed by a 3 mm erosion and 1 mm dilation using the FSL software [47], [48]. The process of erosion and dilation was done to minimize partial volume effects when going from a high resolution NAWM mask to the low resolution T1 measurements. Using a linear transformation, the MPRAGE and a standard MNI template [49] were co-registered, allowing for automatic and robust removal of the volume below the line from the superior surface of the anterior commissure to the center of the posterior commissure (the AC-PC line). This step removed the cerebellum as well as the pre-frontal areas, the latter being subject to main field (B0) inhomogeneities. Finally, the MPRAGE and the T1 measurement were coregistered, and all analyses of the T1 maps were performed in their native space.

Preprocessing in healthy controls: The same procedure was applied to the MRI data sets acquired in healthy subjects except for the steps involving the identification and exclusion of white matter lesions. The mean T1 value and its coefficient of variation (ratio of the standard deviation to the mean) were calculated for the NAWM mask of patients and the white matter mask of healthy subjects. The coefficient of variation provides a measure of variability of the T1 value within the white matter for each individual and was used as an index of T1 heterogeneity of the tissue.

### Statistical Analysis

The mean T1 value as estimated for NAWM in MS patients and cerebral white matter in healthy controls was defined as primary variable of interest. Between-group differences as well as within-subject decreases in T1 value after Gd-DTPA injection were statistically assessed using a two-factorial repeated measure analysis of variance (ANOVA) with the between-subject factor “group” (MS patients vs. healthy controls) and the within-subject factor “time” (before vs. after Gd-DTPA injection). Greenhouse-Geisser correction for non-sphericity was applied conditional on a significant Mauchly’s test and ANOVAs were followed by post-hoc one-sided paired-sample t-tests to assess the decrease in T1 values after Gd-DTPA injection in each group or by two-sided independent-samples t-tests to assess differences in T1-values between groups. An additional ANOVA was performed using the coefficient of variation of the T1 values as dependent variable to assess possible effects of Gd-DTPA injection on the variability of T1 values in the white matter. Multiple regression analysis was performed to test for correlations between EDSS or age and T1 values before as well as after injection of contrast agent. Effect of treatment (interferon vs. glatiramer acetate) was tested by a two-sided independent-samples t-test. P-values <0.05 were considered significant. Data are presented as mean ± SD if not specified otherwise. Statistical analyses were performed using SPSS version 19.
